# 567. Therapeutic Drug Monitoring of Extended Infusion Ceftazidime-Avibactam-Aztreonam (CAZ-AVI-AZT) in Critically Ill Patients with Multidrug-Resistant (MDR) Gram-Negative Infections

**DOI:** 10.1093/ofid/ofaf695.176

**Published:** 2026-01-11

**Authors:** Anas Hakkim, M Pharm, Dipu T Sathyapalan, Merlin Moni, Narmadha M P, Georg Gutjahr, Jahnavi Mohandas, Anil kumar V, Anjana Venugopal, Venugopalan Veena, Zubair Umer Mohamed, Aneesh T P

**Affiliations:** Amrita School of Pharmacy, AIMS Health Sciences Campus, Kochi, Kerala, India., Palakkad, Kerala, India; Professor, Department of Internal Medicine, Lead Division of Infectious diseases, Administrative chair, URUM, chairman HICC, Kochi, Kerala, India; Amrita Institute of Medical Sciences, Kochi, Kochi, Kerala, India; Amrita School of Pharmacy, Kochi, Ernakulam, Kerala, India; Amrita Vishwa Vidyapeetham University, Kochi, Kerala, India; Amrita Institute of Medical Sciences, kochi, Kerala, India; Amrita Institute of Medical Sciences Ponekara, Kochi, Kochi, Kerala, India; Amrita Institute of Medical Sciences, Kochi, Kochi, Kerala, India; University of Florida, College of Pharmacy, Gainesville, Florida; Amrita Institute of Medical Sciences, kochi, Kerala, India; Amrita Vishwa Vidyapeethan, Ernakulam, Kerala, India

## Abstract

**Background:**

Pharmacokinetic variability in critically ill patients complicates β-lactam dosing and increases the risk of treatment failure, particularly in infections caused by pathogens with elevated MICs. The combination of CAZ-AVI-AZT is used to treat complex infections caused by MDR *Enterobacterales*. This study evaluates pharmacokinetic/pharmacodynamic (PK/PD) target attainment of CAZ-AVI-AZT via extended infusions in critically ill patients.

**Figure:**
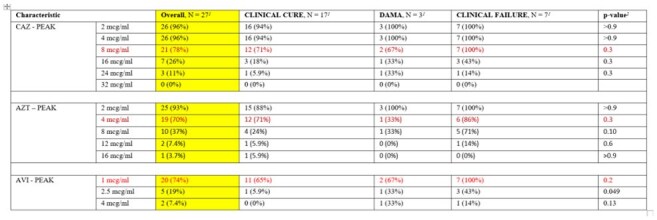
Overall Target Attainment for CAZ-AVI-AZT at Peak Levels (50% fT)

**Figure:**
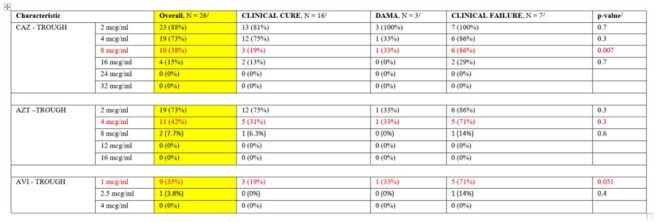
Overall Target Attainment for CAZ-AVI-AZT at Trough Levels (100% fT)

**Methods:**

A prospective, observational study was conducted at a tertiary care hospital in South India from Dec 2023 to Jun 2024, involving critically ill adult patients with confirmed/suspected MDR *Enterobacterales* infections who received ≥ 4 doses of CAZ-AVI-AZT administered via extended infusions (3-hour). Those with GFR < 15 mL/min and receiving dialysis were excluded. The E-strip stacking method was used to confirm that all isolates exhibited synergistic activity to CAZ-AVI-AZT. Clinical and microbiological cure at 30 days and at the end of therapy were also evaluated. PK/PD targets were 50% fT ≥ MIC and 100% fT ≥ MIC (50% free drug time above MIC and 100% free time above MIC, respectively).

Target Attainment Based on CAZ-AVI Susceptibility at Peak Level

**Figure:**
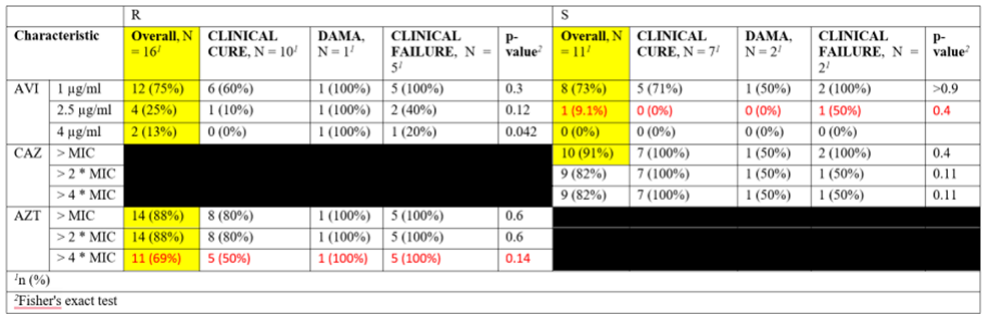

Target Attainment Based on CAZ-AVI Susceptibility at Trough Level

**Figure:**
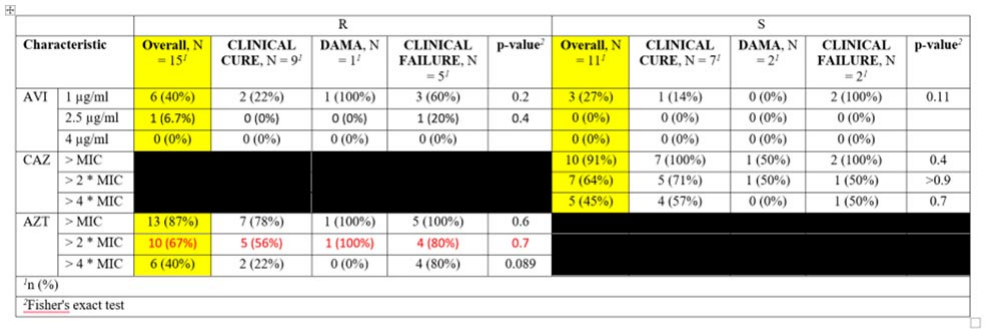

**Results:**

Among 27 patients, the median age was 52 years (43, 61). The most common sources of infection were bacteraemia (37%), pneumonia (30%), UTI (22%), and IAI (11%). The most common pathogens isolated were *K. pneumoniae* (89%) and *E. coli* (11%). Median peaks/troughs (mcg/mL): CAZ 11.9/7.0, AVI 1.24/0.77, AZT 5.7/3.55.

50% fT ≥ MIC (of 8, 1, and 4 µg/mL, respectively) was achieved in 78%, 74%, and 70% of ceftazidime, avibactam, and aztreonam, respectively. 100% fT ≥ MIC was only attained in 38%, 35%, and 42% of ceftazidime, avibactam, and aztreonam, respectively. Only one patient had a microbiological failure, and clinical cure was achieved in 17 (63%) patients. Microbiological and clinical success rates did not vary between those who did or did not attain targets.

**Conclusion:**

Our findings highlight suboptimal drug levels, variability, and lack of target attainment, stressing the need for dose optimization in this high-risk population.

**Disclosures:**

All Authors: No reported disclosures

